# A self-evaluation tool for integrated care services: the Development Model for Integrated Care applied in practice

**Published:** 2012-09-04

**Authors:** Mirella Minkman, Lidewij Vat

**Affiliations:** Vilans, The Netherlands; Vilans, The Netherlands

**Keywords:** development model for integrated care, development of integrated care services, implementation and improvement of integrated care, self evaluation

## Abstract

**Purpose:**

The purpose of the workshop is to show the applications of the Development Model for Integrated Care (DMIC) in practice. This relatively new and validated model, can be used by integrated care practices to evaluate their integrated care, to assess their phase of development and reveal improvement areas. In the workshop the results of the use of the model in three types of integrated care settings in the Netherlands will be presented. Participants are offered practical instruments based on the validated DMIC to use in their own setting and will be introduced to the webbased tool.

**Context:**

To integrate care from multiple providers into a coherent and streamlined client-focused service, a large number of activities and agreements have to be implemented like streamlining information flows and adequate transfers of clients. In the large range of possible activities it is often not clear what essential activities are and where to start or continue. Also, knowledge about how to further develop integrated care services is needed. The Development Model for Integrated Care (DMIC), based on PhD research of Mirella Minkman, describes nine clusters containing in total 89 elements that contribute to the integration of care. The clusters are named: ‘client-centeredness’, ‘delivery system’, ‘performance management’, ‘quality of care’, ‘result-focused learning’, ‘interprofessional teamwork’, ‘roles and tasks’, ‘commitment’, and ‘transparant entrepreneurship’ [1–3]. In 2011 a new digital webbased self-evolution tool which contains the 89 elements grouped in nine clusters was developed. The DMIC also describes four phases of development [4]. The model is empirically validated in practice by assessing the relevance and implementation of the elements and development phases in 84 integrated care services in The Netherlands: in stroke, acute myocardial infarct (AMI), and dementia services. The validation studies are recently published [5, 6]. In 2011 also other integrated care services started using the model [7]. Vilans developed a digital web-based self-evaluation tool for integrated care services based on the DMIC. A palliative care network, four diabetes services, a youth care service and a network for autism used the self-evaluation tool to evaluate the development of their integrated care service. Because of its generic character, the model and tool are believed to be also interesting internationally.

**Data sources:**

In the workshop we will present the results of three studies in integrated diabetes, youth and palliative care. The three projects consist of multiple steps, see below. Workshop participants could also work with the DMIC following these steps.

One: Preparation of the digital self-evolution tool for integrated care services

Although they are very different, the three integrated care services all wanted to gain insight in their development and improvement opportunities. We tailored the digital self-evaluation tool for each specific integrated care services, but for all the basis was the DMIC. Personal accounts for the digital DMIC self-evalution survey were sent to multiple partners working in each integrated care service (4–16 partners).

Two: Use of the online self-evaluation tool each partner of the local integrated care setting evaluated the integrated care by filling in the web-based questionnaire. The tool consists of three parts (A-C) named: general information about the integrated care practice (A); the clusters and elements of the DMIC (B); and the four phases of development (C). The respondents rated the relevance and presence of each element in their integrated care practice. Respondents were asked to estimate in which phase of development their thought their service was.

Three: Analysing the results

Advisers from Vilans, the Centre of excellence for long-term care in the Netherlands, analysed the self-evolution results in cooperation with the integrated care coordinators. The results show the total amount of implemented integrated care elements per cluster in spider graphs and the development phase as calculated by the DMIC model. Suggestions for further development of the integrated care services were analysed and reported.

Four: Discussing the implications for further development

In a workshop with the local integrated care partners the results of the self-evaluation were presented and discussed. We noticed remarkable results and highlight elements for further development. In addition, we gave advice for further development appropriate to the development phase of the integrated care service. Furthermore, the professionals prioritized the elements and decided which elements to start working on. This resulted in a (quality improvement) plan for the further development of the integrated care service.

Five: Reporting results

In a report all the results of the survey (including consensus scores) and the workshops came together. The integrated care coordinators stated that the reports really helped them to assess their improvement strategy. Also, there was insight in the development phase of their service which gave tools for further development.

**Case description:**

The three cases presented are a palliative network, an integrated diabetes services and an integrated care network for youth in the Netherlands. The palliative care network wanted to reflect on their current development, to build a guiding framework for further development of the network. About sixteen professionals within the network worked with the digital self-evaluation tool and the DMIC: home care organisations, welfare organizations, hospice centres, health care organisations, community organizations.

For diabetes care, a Dutch health care insurance company wished to gain insight in the development of the contracted integrated care services to stimulate further development of the services. Professionals of three diabetes integrated care services were invited to fill in the digital self-evaluation tool. Of each integrated care service professionals like a general practitioner, a diabetes nurse, a medical specialist, a dietician and a podiatrist were invited. In youth care, a local health organisation wondered whether the DMIC could be helpful to visualize the results of youth integrated care services at process- and organisational level. The goal of the project was to define indicators at a process- and organisational level for youth care services based on the DMIC. In the future, these indicators might be used to evaluate youth care integrated care services and improve the quality of youth care within the Netherlands.

**Conclusions and discussion:**

It is important for the quality of integrated care services that the involved coordinators, managers and professionals are aware of the development process of the integrated care service and that they focus on elements which can further develop and improve their integrated care. However, we noticed that integrated care services in the Netherlands experience difficulties in developing their integrated care service. It is often not clear what essential activities are to work on and how to further develop the integrated care service. A guiding framework for the development of integrated care was missing. The DMIC model has been developed for that reason and offers a useful tool for assessment, self-evaluation or improvement of integrated care services in practice. The model has been validated for AMI, dementia and stroke services. The latest new studies in diabetes, palliative care and youth care gave further insight in the generic character of the DMIC. Based on these studies it can be assumed that the DMIC can be used for multiple types of integrated care services. The model is assumed to be interesting for an international audience. Improving integrated care is a complex topic in a large number of countries; the DMIC is also based on the international literature. Dutch integrated care coordinators stated that the DMIC helped them to assess their integrated care development in practice and supported them in obtaining ideas for expanding and improving their integrated care activities.

The web-based self-evaluation tool focuses on a process- and organisational level of integrated care. Also, the self assessed development phase can be compared to the development phase as calculated by the DMIC tool. The cases showed this is fruitful input for discussions. When using the tool, the results can also be used in quality policy reports and improvement plans. The web-based tool is being tested at this moment in practice, but in San Marino we can present the latest webversion and demonstrate with a short video how to use the tool and model. During practical exercises in the workshop the participants will experience how the application of the DMIC can work for them in practice or in research. For integrated care researchers and policy makers, the DMIC questionnaire and tool is a promising method for further research and policy plans in integrated care.

## Information about the authors

Mirella Minkman, RN Msc is program leader Quality and Innovation in Elderly Care at Vilans, the Dutch Center of Excellence in long-term care. In January 2012 she will defend her PhD thesis ‘Developing Integrated care’.

Lidewij Vat, Msc is program worker Quality and Innovation in Elderly and Chronically Care at Vilans, the Dutch Center of Excellence in long-term care. She led the development of the web-based tool and is involved in multiple integrated care projects.
